# Design of the Verbiest trial: cost-effectiveness of surgery versus prolonged conservative treatment in patients with lumbar stenosis

**DOI:** 10.1186/1471-2474-12-57

**Published:** 2011-03-03

**Authors:** Gijsbert M Overdevest, Pim AJ Luijsterburg, Ronald Brand, Bart W Koes, Sita MA Bierma-Zeinstra, Just AH Eekhof, Carmen LAM Vleggeert-Lankamp, Wilco C Peul

**Affiliations:** 1 Department of Neurosurgery, Leiden University Medical Center, Albinusdreef 2, 2333 ZA Leiden, The Netherlands; 2 Department of Neurosurgery, Medical Center Haaglanden, Lijnbaan 32, 2512 VA The Hague, The Netherlands; 3 Department of General Practice, Erasmus MC, University Medical Center, 's-Gravendijkwal 230, 3015 CE Rotterdam, The Netherlands; 4 Department of Medical Statistics, Leiden University Medical Center, Albinusdreef 2, 2333 ZA Leiden, The Netherlands; 5 Department of Orthopaedic Surgery, Erasmus MC, University Medical Center, 's-Gravendijkwal 230, 3015 CE Rotterdam, The Netherlands; 6 Department of General Practice, Leiden University Medical Center, Albinusdreef 2, 2333 ZA Leiden, The Netherlands

## Abstract

**Background:**

Degenerative changes of lumbar spine anatomy resulting in the encroachment of neural structures are often regarded progressive, ultimately necessitating decompressive surgery. However the natural course is not necessarily progressive and the efficacy of a variety of nonsurgical interventions has also been described. At present there is insufficient data to compare surgical and nonsurgical interventions in terms of their relative benefit and safety. Previous attempts failed to provide clear clinical recommendations or to distinguish subgroups that substantially benefit from a certain treatment strategy. We present the design of a randomized controlled trial on (cost-) effectiveness of surgical decompression versus prolonged conservative treatment in patients with neurogenic intermittent claudication caused by lumbar stenosis.

**Methods/Design:**

The aim of the Verbiest trial is to evaluate the effectiveness of prolonged conservative treatment compared to decompressive surgery. The study is a multi-center randomized controlled trial with two parallel groups design. Patients (age over 50) presenting to the neurologist or neurosurgeon with at least 3 months complaints of neurogenic intermittent claudication and considering surgical treatment are eligible for inclusion. Participants are randomly allocated to either prolonged conservative treatment, receiving further treatment from their general practitioner and physical therapist, or allocated to surgery and operated within 4 weeks. Primary outcome measure is the functional assessment of the patient as measured by the Zurich Claudication Questionnaire at 24 months of follow-up. Data is analyzed according to the intention to treat principle.

**Discussion:**

With a cost-effectiveness analysis the trade off between the costs of prolonged conservative treatment and delayed surgery in a smaller number of patients are compared with the current policy of surgical management. As surgery is expected to be inevitable in certain subgroups of patients, the distinction of and classification by predictive patient characteristics is most relevant to clinical practice.

**Trial registration:**

Netherlands Trial Register (NTR): NTR2216

## Background

Lumbar stenosis is defined as a reduction in the diameter of the spinal canal, lateral nerve canals, or neural foramina. Most frequently lumbar stenosis is the result of a degenerative disease process and consequently involves multiple levels of the lumbar spine or sites of lumbar stenosis. Degenerative lumbar stenosis is caused by bone hypertrophy, osteoarthritis of facet joints, ligamentous hypertrophy, disc protrusion, spondylolisthesis alone or any combination of these elements. Central canal stenosis results in compression of the cauda equina. Lateral recess stenosis and neuro foraminal stenosis result in encroachment of the nerve root. Degenerative spinal stenosis most commonly affects the L3-L4, and L4-L5 segments to cause cauda equina compression [1,2].

Symptoms related to lumbar stenosis range from numbness, fatigue to actual pain of the buttocks, thighs, and legs. Back pain and muscle weakness are also frequently reported. Symptoms may radiate from the buttocks to the distal lower extremities and are often accompanied with paraesthesias. In contrast to sciatica symptoms are generally bilateral and localized poorly [2]. A pathognomonic aspect of lumbar stenosis is the relation between symptoms and function. Symptoms are typically aggravated by standing and walking. However symptoms generally decrease with sitting or standing with lumbar flexion and with lying. As symptoms worsen patients become progressively limited in their activities and walking distance. This relationship is known as neurogenic intermittent claudication [3]. The clinical entity was first described by the Dutch neurosurgeon Verbiest in 1950, thus formerly also referred to as the Verbiest syndrome [4].

Degenerative lumbar stenosis is the most common reason for lumbar surgery in elderly people [5]. Taking into account that the mean age of the total population of the Netherlands and other Western countries will increase over the upcoming years and due to an increasing life expectancy in general, surgery rates are expected to increase even further. However the aging of the population does not explain the total increase of lumbar surgery rates observed over the past decades. Also there are significant geographic variations in spine surgery rates [5-7]. Despite extensive research in this area, various authors suggest a poor consensus on indications for surgery or the choice of particular surgical procedures. Patient demographic and clinical characteristics may be outweighed by surgeons and patients preferences concerning treatment allocation [5-8].

Although the condition is frequently diagnosed and various surgical and nonsurgical interventions for individuals with neurogenic intermittent claudication are widely accepted in clinical practice, there is limited evidence to support many of them, especially in terms of their relative benefit and risk compared with other options [9-11]. The long-term results of surgical management of spinal stenosis are good or excellent in 50-79% of patients [9]. Most cases of spinal stenosis can be treated with surgical decompression alone. In a minority of cases spinal stenosis is accompanied with a spinal deformity such as severe spondylolisthesis or scoliosis. In these instances the addition of spinal fusion to decompression may be necessary to maintain spinal stability and effectively decompress neural structures [9]. The available evidence suggests no advantage in routinely applying fusion techniques [11].

In a randomized controlled trial (RCT) comparing the effectiveness of early surgery to prolonged conservative treatment among patients with sciatica, early surgery favored a better short-term outcome compared to prolonged conservative management [12]. However, there was no significant nor clinically relevant difference between treatment groups after 1 year. It is well known that the natural history of sciatica caused by lumbar disk herniation is favorable in most cases [13]. Spontaneous involution of herniated disk material can also be observed with imaging studies [14]. By contrast lumbar stenosis is caused by long-term degenerative changes affecting the structures surrounding the cauda equine or nerve roots. Unlike disc herniation these degenerative changes are not likely to resolve spontaneously. However the extent of narrowing of the central or lateral canal is not necessarily associated with symptom severity [2]. In addition, imaging studies demonstrate radiological significant lumbar stenosis can be found in up to 20 percent of asymptomatic elderly individuals [15,16].

Various studies observing the natural course of neurogenic intermittent claudication report improvement of symptoms and conclude that expectant observation could be an alternative to surgical treatment [9,17-21]. In a review by Watters et al. the effectiveness of nonsurgical therapy is discussed [9]. Favorable outcomes are achieved in approximately 70% of patients with moderate symptoms and in 33% with severe symptoms. Approximately 20-40% of patients with moderate symptoms who are treated conservatively require surgery in the long-term (2-10 years). However, overall observational results of surgical treatment consistently demonstrate better short-term and long-term results when compared to conservative therapy. Consequently, and despite the apparent superiority of surgical treatment, nonsurgical treatment can be an effective therapy in selected patients. Furthermore surgical intervention is equally effective when performed after failure of conservative treatment compared to primary surgical management [17].

Limited evidence is available regarding the efficacy for nonsurgical treatments in the treatment of lumbar stenosis [9,10]. Nonsurgical treatments have most frequently been compared to other treatments, rather than to the natural course of lumbar stenosis. Currently the choice of treatment is guided mainly by clinical judgment, observational studies and in analogy to other back conditions. A systematic review assessing the effectiveness of nonsurgical treatment of nonspecific low back pain suggests efficacy for a variety of nonsurgical interventions [10]. Treatment should focus on patient education, medications to control pain and exercise therapy [10,22].

Two previous RCTs comparing the effectiveness of surgery to nonsurgical treatment of degenerative lumbar stenosis have been performed [23,24]. Weinstein et al. reported favorable results for surgery throughout the 2 year follow-up period [23]. However the design of the trial was compromised due to large cross-over rates among both treatment groups, at least if one wants to estimate the true magnitude of the effect of surgery compared to nonsurgical treatment. Cross-over to some extent is to be expected, as a subgroup of patients will have complaints refractory to nonsurgical management. However there was also considerable nonadherence among subjects allocated to surgery; only 67% of the surgical group underwent surgery during the two years of follow-up and non-adherence was even larger at earlier time points (54% at 6 months and 42% at 3 months). Possibly subjects reported significant symptom relief before surgery had been scheduled. More likely, however, these subjects did not have symptoms severe enough to justify surgical treatment or did not yet consider surgery as treatment. To address treatment nonadherence conclusions were based on an as treated analysis rather than an intention to treat analysis. These results must be interpreted cautiously as confounding is no longer controlled by random allocation. Although the analysis was corrected for known confounding factors, baseline comparability for other factors can not be assured. Further, the validity of conclusions is limited due to a patient selection bias since post-randomization eligibility for surgery in the initially conservative group and vice versa is clearly correlated to the condition of the patient prior to the cross-over. Malmivaara et al. and also reported favorable results for surgery as compared to conservative treatment [24]. Based on a previously defined minimal clinically imported difference (MCID) with regard to functional disability 63% of surgically managed subjects reported improvement compared to 30% of conservatively managed subjects [25]. In concordance with previous studies the initial advantage of surgery over conservative management was followed by a narrowing of the relative benefit over time [18,26]. Apart from the relatively small sample size, only subjects with moderate symptom severity were included. Final conclusions were further limited by the heterogeneity of treatments as surgical decompression was accompanied by fusion techniques in 20% of the subjects allocated to surgery. The distinction of surgical candidates in both RCT deserves scrutiny, especially since the generalizability of the findings depends on the ability of the study design to reflect the current clinical challenge.

At present there is still a paucity of high quality evidence to justify the timing of surgery and useful clinical parameters to identify subjects for the most appropriate treatment strategy are lacking. The primary goal of this study is to assess the (cost-) effectiveness of a policy of conventional surgical intervention as compared to prolonged conservative management in the treatment of degenerative lumbar stenosis. Further, patient characteristics and clinical findings are evaluated to define subgroups that substantially benefit from one of the two proposed treatment strategies.

## Methods and design

The study is designed as a multi-center RCT with two parallel groups. The multi-center design is necessary to accrue the required amount of patients and obtain generalizable results. The trial is conducted in the hospitals, which previously collaborated in the Sciatica trial [12]. The medical research ethics committee of each hospital has approved the trial. If inclusion and exclusion criteria are met, patients are asked to participate in the proposed trial. Patients are randomized if the diagnosis lumbar stenosis is confirmed by imaging findings and an indication for surgery is confirmed by the neurosurgeon. Participants are allocated to either prolonged conservative treatment or surgery. As national consensus guidelines for the nonsurgical treatment of lumbar stenosis are lacking a clinical treatment protocol was developed for this trial. The purpose of such a protocol is to provide comparable nonsurgical treatment throughout the participants and aid adherence to treatment allocation. Surgery is scheduled within 4 weeks after randomization. As the clinical investigator and the research nurse offer counseling and evaluate possible negative health effects of treatment allocation, they cannot be blinded for randomization outcome. Throughout the study period participants fill out questionnaires and physical examination findings are recorded during consecutive visits to the outpatient clinic. The follow-up period will last 5 years. After six months secondary surgery is offered in case prolonged conservative treatment does not result in satisfactory symptom relief or functional improvement.

### Participants and recruitment

Patients presenting to the neurologist or neurosurgeon in the participating hospitals with at least 3 months complaints of neurogenic intermittent claudication and considering surgical treatment are eligible for inclusion. The duration of these complaints is one of the main (a priori defined) factors to be evaluated for a possible treatment-effect-modification. Inclusion and exclusion criteria are listed in Table 1.

**Table 1:** Inclusion and exclusion criteria.

Inclusion criteria	
	- Age ≥50 years old
	- ≥3 Months neurogenic intermittent claudication, as noted by leg/buttock/groin pain with or without back pain *or *fatigue in the legs provoked by walking. Leg/buttock/groin pain or fatigue needs to be relieved by lumbar flexion
	- Narrowed lumbar spinal canal, nerve root canal or intervertebral foramen at one or more levels confirmed by MRI
	- Regular indication for surgical decompression
	- Signed informed consent

**Exclusion criteria**	

	- Cauda equine syndrome
	- Paget's disease, severe osteoporosis or metastasis to the vertebrae
	- Significant scoliosis (Cobb angle > 25 degrees)
	- Previously laminectomy at the same level, has degenerative or lytic spondylolisthesis ≥ grade 2 (on a scale 1 to 4) at the affected level or significant instability of the lumbar spine
	- Severe comorbid conditions that increase the risk to the patient or interfere with the evaluability of this study (e.g. severe ischemic heart disease, musculoskeletal or neurological conditions impairing walking ability, cognitive impairment (MMSE <25 points)
	- Unable to read or write Dutch

General practitioners and physical therapists in the vicinity of the participating hospitals have been informed at the start of the trial. They are directly involved as they provide primary care for subjects allocated to prolonged conservative treatment. Secondly, general practitioners are asked to refer potentially eligible patients with neurogenic intermittent claudication to the outpatient clinics for evaluation by the neurologist.

### Prolonged conservative management

Conservative management is a prolonged nonsurgical treatment policy conducted by the general practitioner. The general practitioner provides additional information about the condition's causes, symptoms and treatment options. Further, the efficacy of the prescribed pain medication is reviewed and if necessary alterations are made, taking into account possible contra-indications in the elderly. Subjects are advised to stay active and if possible return to work and/or their leisure activities. The general practitioner prescribes physical therapy, which consists of active exercises to guide the patient in upgrading his or her activities according to an agreed time schedule. The guide is time, not the intensity of the pain. The protocol for the individual exercise therapy supervised by a physical therapist includes education, stretching, strengthening and conditioning exercises [27]. Education in proper posture, body mechanics for daily activities, and if necessary, the use of orthopedic aids is essential to maintain the gains made through the physical therapy sessions. The goal of stretching and strengthening is to decrease the extension forces on the lumbar spine attributable to agonist muscle tightness, antagonist weakness or both, which results in a decreased lumbar lordosis. Stretching exercises include hip flexor stretching, hamstring stretching and lumbar paraspinal stretching. The strengthening exercises primarily target the abdominal and lower extremity muscles. The conditioning exercises include walking on a treadmill, on level and inclined surfaces and/or riding a stationary bicycle, with the goal of increasing general fitness. A maximum of 9 sessions is allowed in the first 3 months. Three additional booster sessions take place in the fourth, fifth, and sixth month [10]. A physical therapy session in primary care lasts about 30 minutes. The exercise therapy is discontinued if, according to the physical therapist, treatment goals have been achieved.

### Surgical management

After induction of general endotracheal anaesthesia the patient is positioned prone or in knee-chest position. The level on which to operate is determined through anatomical landmarks and confirmed by fluoroscopy. After a midline posterior skin and subcutaneous tissue incision centered at the interspinous level to be decompressed, the dissection goes through the dorsolumbar fascia. The multifidi are detached from both sides of the spinous processes and laminar attachments. Paraspinal muscles are than retracted laterally. The interspinous ligament is resected to enable the removal of the inferior aspect of the cranial lamina and the superior aspect of the caudal lamina. Subsequent a flavectomy is performed. The decompression is extended laterally with an undercutting of the medial aspect of the facet joints and if necessary an extensive foraminotomy is performed. In case of severe bony stenosis a complete laminectomy is performed. No transpedicular fixation of the spine is performed during the first surgery. Hospital admission is 2 to 4 days, including the day of surgery. During the post-operative period the patients are mobilized accompanied by a physical therapist, starting 3 hours after surgery. If the patient regains the physical ability to manage basis activities of daily living, the patient is discharged. At home the patients visit their own physical therapist who guides them and provides exercises. If possible patients gradually resume their daily activities and work if applicable. The patient is allowed to resume all activities in case no physical limitations are present.

### Study measurements

Clinical data is collected during consecutive follow-up visits to the outpatient clinics at baseline and at 12, 26, 52, 104, 260 weeks after randomization. Subjects fill in questionnaires at selected time points which are returned during the outpatient clinic visits or by mail (Table 2). Demographic data, physical examination findings information on pain intensity, imaging findings, illness related disability, societal costs and utilities and quality of life are collected. Further, subjects keep a diary on the financial consequences and health care consumption related to lumbar stenosis. Visits to the general practitioner, physical therapist, medical specialists, alternative health practice, use of analgesics, duration of sick leave from work and additional utility/mobility costs are recorded. The patient diaries are returned to the research nurse during the outpatient clinic visits or by mail.

**Table 2:** Study measurements over time.

Time in weeks	-2	0	4	12	26	38	52	104	156	208	260
Inpatient visit: surgery			x								
Outpatient visit	x	x		x	x		x	x			x
Questionnaire		x	x	x	x	x	x	x	x	x	x

Patient demographics	x										
MMSE	x										
Basic physical examination	x										
Neurological examination	x			x	x		x	x			x
X-ray	x		x								
MRI	x										
ZCQ		x	x	x	x	x	x	x	x	x	x
Shuttle Walking Test		x			x		x	x			x
Accelerometry		x			x		x	x			x
SPPB		x		x	x		x	x			x
RMDQ		x	x	x	x	x	x	x	x	x	x
SF-36		x	x	x	x	x	x	x	x	x	x
Perceived Recovery			x	x	x	x	x	x	x	x	x
VAS for legs and back		x	x	x	x	x	x	x	x	x	x
EuroQol-5D		x	x	x	x	x	x	x	x	x	x
Patient diary				x	x		x	x	x	x	x
Complications			x	x	x		x	x			x
Re-operation			x	x	x		x	x			x

MMSE: mini-mental state examination; ZCQ: Zurich claudication questionnaire; SPPB: short physical performance battery; RMDQ: Roland Morris disability questionnaire; SF-36: short form 36; VAS: visual analogue scale.

### Primary outcome measure

#### Zurich Claudication Questionnaire (ZCQ)

The ZCQ is a validated disorder-specific outcome measure specifically designed for lumbar spinal stenosis [28,29]. The questionnaire consists of 3 scales: a symptom severity scale, a physical performance scale and a patient satisfaction scale. Multiple validated questionnaires exist for the use in evaluating back conditions, however the ZCQ specifically addresses the symptoms and functional deficits associated with spinal stenosis. Further, this questionnaire provides additional information on patient's satisfactory with the results of surgery.

### Secondary outcome measures

#### Shuttle Walking Test (SWT)

The functional status of the patient can be assessed by the SWT. The SWT has previously been used as an outcome measure in the evaluation of patients with neurogenic intermittent claudication [29]. Subjects are required to walk back and forth, turning around two cones placed 9 meters apart making the shuttle distance 10 meters long. The distance travelled during 15 minutes and/or the time until neurogenic intermittent claudication prohibits further walking is recorded.

#### Mobility examination

Allocation to prolonged conservative treatment may possibly result in subjects becoming less active. As the activity level decreases, situations as walking/standing that give rise to symptoms of neurogenic intermittent claudication diminish and possibly result in symptom/pain relief. Although physical activity, disability and symptom severity are assessed during follow-up examinations, typically the reliability and validity of the measurement of habitual physical activity by questionnaires is low [30]. A continuous 7-day measurement with triaxial accelerometers (GENEA^®^, Unilever) provides information about the total amount, the frequency, the intensity, and the duration of physical activity [31-33]. Further, a common geriatric assessment, the Short Physical Performance Battery (SPPB), is used to assess how well older persons perform simple movements that represent the building blocks of daily activities that require good lower extremity function [34].

#### Roland Morris Disability Questionnaire (RMDQ)

The RMDQ is one of the most frequently used functional disability questionnaires for back-related conditions. The RMDQ was originally designed as a back condition specific questionnaire but is also widely used for patients with sciatica and neurogenic intermittent claudication [18,35,36]. The RMDQ score ranges from 0 (no disability) to 23 (maximum disability). A validated Dutch language version of the RMDQ is available. Compared to the Oswestry disability, of which no validated Dutch version is available, the sensitivity to change is in favor of the RMDQ [37].

#### Short Form-36 (SF-36)

The SF-36 is generic health survey questionnaire that measures overall health status, functional status and health-related quality of life [38,39]. The questions are divided in to eight domains: physical functioning, physical role limitations, emotional role limitations, social functioning, physical pain, general mental health, vitality, general health perception. For each domain, item scores are coded, summed, and transformed on to a scale from 0 (worst health) to 100 (best health).

#### Perceived Recovery

Perceived recovery is assessed with a seven-point likert scale varying from 'completely recovered' to 'worse than ever'. The scales are completed by the patient, the research nurse and the neurosurgeon.

#### Visual analogue scale (VAS) for pain in back and leg

The pain intensity experienced by the patient during the past week is assessed on a horizontal 100 millimeters scale varying from 0 millimeter, "no pain", to 100 millimeters 'the worst pain I can imagine' [40,41]. Back and leg pain is assessed separately.

#### Societal costs and utilities

Cost-effectiveness is expressed as cost per QALY, based on the QALY difference between the surgery and prolonged conservative treatment group. QALYs are estimated by using mean health state values during consecutive follow-up assessments with the EuroQol-5 D. The EuroQol-5 D is a generic health related quality of life questionnaire frequently used for investigating cost-effectiveness [42,43]. Treatment costs and direct medical costs are estimated on basis of the cost centre method. Participants are requested to record direct medical costs (e.g. physiotherapy, visits to general practitioners and medical specialists, nursing care, and medication) and indirect cost (e.g. disability related loss of productivity and additional travel expenses) using a cost diary.

#### Complications and re-operation incidence

The neurosurgeon and research nurse systematically record direct and indirect surgical complications and re-operation incidence. In case of failure of prolonged conservative management considerations for delayed surgery are reviewed.

### Sample size

The success rate of both treatments is determined through perceived recovery and by applying the concept of MCID on the self reported functional disability scores. Derived from previous studies the estimated success rate of surgical management is 60% [9,24,44] and conservative management 40% [9,18,20,24] at 2 years of follow-up. The sample size calculation is based on the comparison of two independent proportions and contains the following parameters: Alpha: 0.05 (2-sided), Beta: 0.10 (90% power), success rate surgery: 0.6, success rate prolonged conservative treatment: 0.4. These assumptions yield a required sample size of 260 patients. Based on an expected drop-out rate of around 10% 280 patients need to be accrued (i.e. 140 patients in each treatment group). A sample size calculation based on the RMDQ yields approximately the same result. A difference of 3 points has been recommended for sample size calculations for clinical trials [36,45]. With Alpha: 0.05, Beta 0.10, μ difference of 3, and a standard deviation of RMDQ scores of 7.6 [18,36] a sample size of 270 patients is required.

### Statistical analysis

Differences between treatment groups at baseline are assessed using univariate analyses to check for a balanced randomization. In case of substantial differences that induce confounding of the main treatment effect, both an uncorrected and a corrected (multivariate) analysis are presented. Data analysis is performed according to the intention to treat principle. The primary hypothesis using the primary outcome measure is a comparison of the average ZCQ over a period of 24 months. All continuous outcome measures are assessed with a repeated-measures analysis of variance with a first-order autoregressive covariance matrix. Differences between treatment groups are assessed by computing both the main effect of the treatment and the interaction between treatment and time in a mixed linear model. The area under curve for the ZCQ scores and secondary treatment outcome variables during the follow-up period are compared both implicitly using the repeated measurements approach. A Kaplan-Meier survival analysis is used to estimate the proportion of patients reporting recovery as a function of time elapsed from randomization. The average difference is characterized by a Hazard Ratio derived from a Cox Proportional Hazards model and the accompanying likelihood ratio test.

Table 3 lists the predefined patient characteristics and clinical findings possibly related to treatment outcome. These patient characteristics and clinical findings are assessed in a series of multivariate models where each particular covariate (patient characteristic or clinical finding) is tested for treatment-effect modification by extending the main analysis models (either the repeated measurements models or the survival models) by the main effect and the treatment*covariate interaction, thus providing a formal testing framework.

**Table 3:** Predefined patient characteristics and clinical findings possibly related to treatment outcome.

Patient characteristics	
	- Age
	- Gender
	- Pain intensity
	- Duration of symptoms
	- Predominant symptom (back pain compared to leg pain)
	- Walking distance
	- Patient's preference for treatment

**Clinical findings**	

	- One versus multiple level stenosis
	- Extent of stenosis (diameter/surface area MRI)
	- Localization stenosis (lateral recess or central canal)
	- Concomitant spondylolisthesis
	- Neurological impairment (muscle weakness, sensory loss)

One covariate is pre-specified as a primary effect-modifier: duration of complaints at randomization. This covariate is clinically crucial since it allows the estimation of a possible "timing-of-surgery" effect by using the natural variability among patients with respect to the duration of complaints at the moment of the decision to perform surgery or to postpone it. Therefore each of the main analyses is accompanied with an extension to test for "duration-of-complaints" * "treatment" interaction. This interaction is tested at the nominal 5% level within the primary analysis. If the interaction is not significant, the primary analysis of this trial defaults to the standard model with main effects. If the interaction is significant, it follows that a fortiori a treatment effect exists (and it varies with the duration of complaints). The model without the interaction still gives the overall (average) effect of the main hypothesis with its associated confidence interval. For the remaining 11 variables in Table 3 a 10% level of significance is applied, due to the reduced power of these tests. First two models are fitted, one with all patient characteristics and their interactions, and one with all clinical factors and their interactions. The total model improvement by adding the set of interactions is tested with the appropriate degrees of freedom. Hence 2 statistical tests are performed and a Bonferroni correction to the alpha level of 5% is applied to test whether any characteristic or clinical finding exists which modifies the treatment effect. When significance in one or both models exists, each individual covariate is tested, using a Bonferroni-Holm correction based on the number of covariates in the respective collection of covariates. Any such factor eventually classified as a significant effect modifier (Bonferroni-Holm with 10% as a starting level of significance) leads to subgroup estimation of treatment effects, to be tabulated in the final analysis.

Data is stored via the internet-based secure data management system "ProMISe" of the department of Medical Statistics and Bio Informatics. The analyses are carried out using appropriate statistical software (e.g. SAS, SPSS).

## Discussion

It has been previously suggested that conservative management should be the initial treatment of degenerative lumbar stenosis and decompressive surgery is appropriate to consider only in case of intolerable pain or disability. Despite these recommendations the overall observed clinical improvement after surgery is disappointing and a substantial proportion of surgical candidates managed conservatively may still report improvement. Taken in to account the unexplained large geographic variation of surgery rates for lumbar stenosis and the long-term narrowing of the relative benefit of surgery compared to conservative management, another critical appraisal of indications for surgery is necessary.

## Abbreviations

ITT: intention to treat; MCID: minimal clinically important difference; MMSE: mini-mental state examination; RMDQ: Roland Morris disability questionnaire; MRI: magnetic resonance imaging; QALY: quality-adjusted life year; RCT: randomized controlled trial; SF-36: short form 36; SPPB: short physical performance battery; SWT: shuttle walking test; VAS: visual analogue scale; ZCQ: Zurich claudication questionnaire.

## Competing interests

The authors declare that they have no competing interests.

## Authors' contributions

GO is the coordinator and principal investigator and drafted the manuscript. WP, PL, BK, SB and RB designed the study protocol. RB is responsible for the statistical analysis and data storage using the ProMISe data management system. CV is involved in the implementation of the trial in the participating hospitals, PL and SB consulted on the protocol for physical therapy, JE consulted on the protocol for the general practitioners. All authors participated in the study design and coordination. All authors read and approved the final manuscript.

## Pre-publication history

The pre-publication history for this paper can be accessed here:

http://www.biomedcentral.com/1471-2474/12/57/prepub
